# Editorial: Molecular Mechanisms of Selective Autophagy in Human Disease

**DOI:** 10.3389/fcell.2020.00664

**Published:** 2020-08-06

**Authors:** Valentina Cianfanelli, Paolo Grumati, Francesca Nazio

**Affiliations:** ^1^Unit of Cell Stress and Survival, Center for Autophagy, Recycling and Disease (CARD), Danish Cancer Society Research Center, Copenhagen, Denmark; ^2^Telethon Institute of Genetics and Medicine, Cell Biology and Disease Mechanisms, Pozzuoli, Italy; ^3^Department of Pediatric Hemato-Oncology and Cell and Gene Therapy, IRCCS Bambino Gesù Children's Hospital, Rome, Italy

**Keywords:** cancer, molecular mechanism, autophagy receptors, human disease, selective autophagy

Autophagy is an essential catabolic process involved in the removal of cytosolic contents through double-membrane vesicles named autophagosomes. Although it has long been considered a bulk non-selective process, it is now clear that autophagy is a highly regulated and specific degradation pathway for the removal of different cellular components. Several forms of selective autophagy have been characterized, such as: mitophagy, ribophagy, ER-phagy, virophagy, pexophagy, aggrephagy, lipophagy, and glycophagy (Kirkin and Rogov, 2019). Recent studies have revealed an intrinsic connection between selective autophagy and human diseases including infections, neurodegenerative disorders and cancer (Levine and Kroemer, 2019). However, our understanding of the regulation and role of selective-autophagy, in distinct diseases, is still in its infancy. This Research Topic aimed at summarizing recent findings on the involvement of selective types of autophagy in different human disorders.

Beese et al. eloquently review the role of three peculiar and probably interconnected types of selective autophagy (ribophagy, ER-phagy, and proteaphagy) in health and disease. Although some regulators and functional consequences of ER-phagy have been discovered (Grumati et al., 2018), the physiological and pathological roles of both ribophagy and proteaphagy are only beginning to be documented. Both ribosome and proteasome degradation is enhanced in stressful conditions and seems to be important for amino acids or nucleotides replenishment. Regarding ER-phagy, a second review by D'Eletto et al. fully describe the importance of this selective form of autophagy in different human disorders such as neuropathies, virus infections and cancers, underling the potential of ER-phagy regulators as novel therapeutic targets.

Of note, computational structural biology is emerging as a useful tool to comprehend the specific roles of mATG8-binding proteins. Sora et al. summarize the methods that help to understand how distinct mATG8s achieve substrate specificity and bind to the membrane lipids. Moreover, computational structural biology could predict the mATG8s conformational ensemble following, for example, post-translational modifications.

Although the importance of autophagy in cancer is well-established (Rybstein et al., 2018), the roles of selective forms of autophagy are not completely characterized yet. Putyrski et al. explore the relationship between selective autophagy and chemotherapy sensitivity in acute myeloid leukemia (AML). In this original article, they use a protein engineering approach to inhibit the LC3 interacting regions (LIRs) of three selective autophagy receptors: OPTN, p62, and NDP52. They found that simultaneous inhibition of the three LIR motifs is sufficient to sensitize the cells to cytarabine, the first-line therapy for AML. Hence, this study suggests the proteins involved in selective autophagy as promising drug targets to restrain AML proliferation.

In a different cancer model, the B cell chronic lymphocytic leukemia (B-CLL), Onnis et al. review the role of the pro-oxidant adaptor protein p66SHC in the regulation of selective autophagy of the B cells. Interestingly, p66SHC acts as a new LC3 mitophagy receptor (Onnis et al., 2018) and emerges as a fundamental regulator of B cell survival and differentiation.

It is also intriguing how autophagy impacts on development of cancer caused by oncogenic human viruses. In a review, Vescovo et al. summarize the crucial role of autophagy during viral infections and how it impacts on cancer growth. Autophagy directly targets viruses for elimination (virophagy). To date, seven oncogenic viruses have been described to hijack autophagic machinery ensuring their endurance and reproduction. The resulting autophagy inhibition could contribute to tumorigenesis because of inefficient cell quality control in the course of infection.

In addition to the role in cancer, a review by Adornetto et al. and an original article by Intartaglia et al. describe the role of autophagy in retinal degeneration. Firstly, Adornetto et al. discuss the controversial role of autophagy in retinal ganglion cells as a pro-survival or pro-death mechanism. This dual role could depend on the dynamicity of the autophagy process and/or on the action of different selective types di autophagy. Intartaglia et al. found an impairment of autophagy flux and an increase in the protein level of the autophagy receptor Nrb1 in a Mucopolysaccharidosis type IIIA mouse model. This supports the idea that an autophagy defect contributes to apoptotic cell death and inflammatory processes in this context. Of note, this finding may have important therapeutic implications for mucopolysaccharidoses.

We hereby thank a lot all the authors that participated in this Research Topic. Their articles significantly contribute to a more comprehensive over-view of the role of different forms of autophagy in human diseases, suggesting potential novel therapeutic strategies too.

## Author Contributions

FN conceived and wrote the Editorial. VC and PG reviewed and edited the manuscript. All authors contributed to the article and approved the submitted version.

## Conflict of Interest

The authors declare that the research was conducted in the absence of any commercial or financial relationships that could be construed as a potential conflict of interest.
